# Validation of Machine Learning-Based Assessment of Major Depressive Disorder from Paralinguistic Speech Characteristics in Routine Care

**DOI:** 10.1155/2024/9667377

**Published:** 2024-04-09

**Authors:** Jonathan F. Bauer, Maurice Gerczuk, Lena Schindler-Gmelch, Shahin Amiriparian, David Daniel Ebert, Jarek Krajewski, Björn Schuller, Matthias Berking

**Affiliations:** ^1^Department for Clinical Psychology and Psychotherapy, Friedrich-Alexander-Universität Erlangen-Nürnberg, 91052 Erlangen, Germany; ^2^Chair of Embedded Intelligence for Health Care & Wellbeing, University of Augsburg, 86159 Augsburg, Germany; ^3^Department for Sport and Health Sciences, Technical University Munich, 80992 Munich, Germany; ^4^Rhenish University of Applied Science Cologne, 50676 Cologne, Germany; ^5^Group on Language, Audio, & Music, Imperial College London, London SW7 2AZ, UK

## Abstract

New developments in machine learning-based analysis of speech can be hypothesized to facilitate the long-term monitoring of major depressive disorder (MDD) during and after treatment. To test this hypothesis, we collected 550 speech samples from telephone-based clinical interviews with 267 individuals in routine care. With this data, we trained and evaluated a machine learning system to identify the absence/presence of a MDD diagnosis (as assessed with the Structured Clinical Interview for DSM-IV) from paralinguistic speech characteristics. Our system classified diagnostic status of MDD with an accuracy of 66% (sensitivity: 70%, specificity: 62%). Permutation tests indicated that the machine learning system classified MDD significantly better than chance. However, deriving diagnoses from cut-off scores of common depression scales was superior to the machine learning system with an accuracy of 73% for the Hamilton Rating Scale for Depression (HRSD), 74% for the Quick Inventory of Depressive Symptomatology–Clinician version (QIDS-C), and 73% for the depression module of the Patient Health Questionnaire (PHQ-9). Moreover, training a machine learning system that incorporated both speech analysis and depression scales resulted in accuracies between 73 and 76%. Thus, while findings of the present study demonstrate that automated speech analysis shows the potential of identifying patterns of depressed speech, it does not substantially improve the validity of classifications from common depression scales. In conclusion, speech analysis may not yet be able to replace common depression scales in clinical practice, since it cannot yet provide the necessary accuracy in depression detection. This trial is registered with DRKS00023670.

## 1. Introduction

Major depressive disorder (MDD) is a leading cause of disability worldwide [1] characterized by symptoms of depressed mood, loss of motivation, and behavioral alterations such as reduced activity and disturbed sleep [2]. Psychotherapeutic and pharmacotherapeutic interventions as well as their combination have been shown to be effective treatments for MDD (e.g., [3]). However, various studies have found high relapse rates after these treatments (e.g., [4]). Thus, there is a significant need to closely monitor health status after acute treatment and to respond quickly with follow-up interventions if sustained remission is not achieved [5–7].

Such monitoring is not only likely to improve patients' health status but also facilitates the efficient use of resources of the healthcare system. Additionally, monitoring-based feedback can help patients become aware of changes in their health status and, hence, of factors anteceding deterioration [8]. As not all patients have regular access to clinical care providers, remote monitoring has become particularly relevant [9]. Empirically, various studies provide evidence that systematic symptom monitoring improves the efficacy of both pharmacotherapy and psychotherapy [10–13]. Monitoring approaches can focus on the assessment of dimensional variables (e.g., depressive symptom severity), diagnostic status of mental illness (e.g., MDD), or both [14]. For monitoring of patients in routine clinical care, the assessment of diagnostic status may be particularly relevant due to its high value for clinical practice [15], since diagnoses determine the allocation of resources of public healthcare systems, support clinical decision-making, and inform patients about their current health status.

To monitor depressive symptom severity and diagnostic status of MDD, typically, self-report measures such as the depression module of the Patient Health Questionnaire (PHQ-9 [16]) or the Beck Depression Inventory II (BDI-II [17]) as well as clinical interviews such as the Structured Clinical Interview for DSM (SCID [18]) or the Hamilton Rating Scale for Depression (HRSD [19]) are used. As the administration and evaluation of these measures requires significant time and effort, patients and therapists alike are likely to underutilize systematic monitoring. Consequently, patient dropout rates are reported to be as high as 30% at follow-up assessments [20], and less than 20% of practitioners utilize systematic symptom monitoring in their treatment of MDD [11]. Thus, there is a need for more user-friendly methods of assessing diagnostic status and symptom severity after and during treatment for MDD. Ideally, such methods assess indicators of MDD continuously, automated, and without interfering with a patient's current activity. Therefore, recent developments in the fields of sensor technology appear to have great potential for long-term monitoring. Preliminary evidence indicates that MDD can be assessed with the help of sensor data on mobility [21], movement [22], gait [23], facial expressions [24], and paralinguistic speech characteristics [25].

Paralinguistic speech characteristics include nonverbal, vocal features of speech such as voice quality, prosody, or resonance. As the speech apparatus is a highly complex muscular structure, it can be hypothesized that the physiological, neurofunctional, and cognitive changes associated with MDD lead to specific changes in such speech parameters [25]. As speech can be recorded ambulatory, at low cost, nonintrusively, and without the necessity of identifying speech content, it appears to be a promising target for long-term monitoring of depression (e.g., by extracting parameters from patients' phone calls or verbal interactions with a mental health app). Empirically, it was shown that speech sampled from individuals meeting criteria for MDD is characterized by various specific parameters such as increased pause duration, reduced speaking rate, and reduced pitch variability [26–28]. In addition, it was shown that changes in speech parameters were shown to coincide with changes in depressive symptom severity [28–30].

Subsequent works have used paralinguistic speech processing (PSP) algorithms that applied machine learning in order to combine a multitude of speech parameters and thereby improve the assessment of depressive symptom severity (e.g., [31–35]) and diagnostic status of MDD (e.g., [36–38]). Machine learning enables the development and utilization of complex, nonlinear algorithmic models that have been trained to predict output variables (“labels,” e.g., diagnostic status) by a large number of input variables (“features,” e.g., speech parameters; see [25]). Empirically, PSP-based machine learning systems classifying diagnostic status of MDD from speech recordings reached maximum balanced accuracies (i.e., mean of sensitivity and specificity) ranging from 67% up to 91% in previous studies [36–57]. It should be noted that there is considerable variability in methodology between studies that results from differences in feature extraction methods, feature selection, machine learning models, recording setups and settings, speech sampling tasks, operationalization of MDD assessments, and general sample characteristics, such as sample size and diagnostic status of speakers. There are several different approaches that aim at optimally modeling speech that entails data relevant for classifying depressed speech. Studies using supervised algorithms, in which previous knowledge guides speech feature selection and the choice of prediction model, achieved maximum balanced accuracies between 69% and 91% (e.g., [38, 40, 45]). Studies using unsupervised deep learning algorithms that apply image classification of spectrograms to extract meaningful patterns independent of prior knowledge achieved maximum balanced accuracies between 67% and 88% and have been argued to be more generalizable [51, 53–56]. Organized research efforts such as the Audio/Visual Emotion Challenge and Workshop series (e.g., [33, 34]) generated a substantial amount of studies that presented PSP-based machine learning approaches developed under comparable conditions by providing researchers with datasets and predefined goals. Furthermore, most studies (e.g., [38, 51, 52]) used preexisting datasets such as the distress analysis interview corpus (DAIC [58]) or the audio-visual depressive language corpus (AViD [59]) that were recorded in laboratory settings. Fewer studies exist on speech recorded in naturalistic settings (e.g., [44, 54, 57]), such as telephone recordings from the dataset collected by Mundt et al. [28] or smartphone recordings from the SH2 corpus [53]. The importance of recording setting is emphasized by a study by Huang et al. [36] where the same machine learning approach showed 19% less accuracy in naturalistic speech samples compared to its application to laboratory speech samples. Furthermore, different approaches to sampling speech have been applied, such as the use of phonetic tasks and reading tasks (e.g., SH2 corpus), free speech tasks (e.g., DAIC-WOZ corpus), or dyadic interactions, such as family interactions (e.g., [49]) or an interview with a clinician (e.g., [42]). Alghowinem et al. [39] and Jiang et al. [47] showed slight superiority of training PSP systems with free speech recordings compared to recordings of read speech for classifying MDD. Lastly, while most studies used speech recordings from the general population, only few studies used speech from patients during or after treatment with maximum balanced accuracies ranging from 67% to 77% for classifying current diagnostic status of MDD [42–44, 57]. However, these studies classifying diagnostic status from speech samples over time are limited by small sample sizes including only between eight [43] and 35 participants [44, 57]. Furthermore, a majority of studies used MDD labels determined by cut-off scores from single questionnaires such as the PHQ-9 and the BDI-II (e.g., [36, 38, 51]), although the SCID is considered to be the present state-of-the-art for diagnosing MDD [60]. The few studies that used clinical interviews are limited by small sample sizes [40, 42–44]. In summary, there are various studies showing significant proof-of-concept for PSP systems classifying MDD from speech. However, the focus on optimizing performance of PSP systems in previous studies may have resulted in a neglect to assure external validity and to evaluate clinical applicability of such models. Consequently, it is unclear whether such machine learning-based PSP systems can also be developed from naturalistic samples for the purpose of monitoring and are applicable for implementation in the context of routine clinical care.

To overcome these limitations, the primary goal of the present study was to evaluate the validity of PSP-based assessment of MDD in the context of routine clinical care. In this study, we investigated to what extent diagnostic status, as assessed with telephone-based clinical interviews, could be predicted by machine learning-based analysis of speech sampled from clinical interview recordings in a large sample of patients who were currently in inpatient treatment for MDD or had recently been discharged. We also aimed to examine possible effects of gender or age on classifications of the PSP system and to compare its accuracy to that of commonly used clinical measures. Finally, we investigated whether the validity of classifications can be improved by developing a machine learning system trained on both speech and depression scales compared to classifications based on depression scales alone.

## 2. Method

### 2.1. Design

To validate the PSP-based automated assessment of MDD, we used data from telephone-based interviews conducted with individuals currently receiving inpatient treatment for MDD as well as after discharge. The interviews were part of a multicenter, three-armed, investigator-blinded randomized controlled clinical trial comparing the effectiveness of a web-based treatment developed to maintain treatment gains after inpatient treatment for depression (project “MasterMind”; for details, see [61]). Interviews were conducted during inpatient treatment and at follow-ups of 3, 6, and 12 months. Data collection took place between January 2013 and October 2015. Since time points and study conditions were irrelevant to the objective of the present study, data was pooled across time points and conditions. The study was approved by the ethics board of the Friedrich-Alexander-Universität Erlangen-Nürnberg (18-73-B). The study's hypotheses were preregistered in the German Clinical Trials Register (Deutsches Register Klinischer Studien, ID: DRKS00023670) on January 17, 2021.

### 2.2. Participants

Participants were recruited from ten inpatient clinics specialized in the treatment of mental disorders in Germany. Participants were included if they were at least 18 years old, had a current or former (<6 months) MDD diagnosis according to DSM-IV (assessed with SCID [62]), had access to the Internet, had sufficient command of the German language, and were able and willing to provide informed consent. Participants were excluded if they had a current diagnosis of any substance disorder (except for nicotine), psychotic disorder, bipolar disorder, or severe cognitive impairment. From the *n* = 472 participants participating in the original study, telephone recordings were available from a subsample of *n* = 267. On average, each participant provided interview data from 2.1 assessment points (SD = 0.9), amounting to a total of 550 telephone recordings. Clinical data from HRSD, QIDS-C, and PHQ-9 was used for validation. The slight majority of the entire sample (59%) was women. The mean age was 45.6 years (SD = 10.5, range = 18-67). At least one comorbid disorder was diagnosed in 42.3% of participants (chronic depression: 10.9%, somatoform disorders: 10.5%, panic disorder with or without agoraphobia: 10.5%, social anxiety disorder: 6.4%, dysthymic disorder: 4.1%, posttraumatic stress disorder: 3.4%, eating disorder: 2.6%, generalized anxiety disorder: 2.6%, and obsessive-compulsive disorder: 1.5%).

### 2.3. Materials

#### 2.3.1. Diagnostic Status

The presence/absence of a diagnosis of MDD was assessed with the Structured Clinical Interview for DSM-IV Axis I disorders (SCID [62]) which was state-of-the-art at the time the study was conducted.

#### 2.3.2. Depression Scales

To additionally validate results, we used a modified version of the HRSD [63], the Clinician-Rated Quick Inventory of Depressive Symptomatology (QIDS-C [64]), and the PHQ-9 [16]. Cut-off scores were used to determine diagnostic status from the 17-item HRSD (cut‐off > 7), the QIDS-C (cut‐off > 5), and the PHQ-9 (cut‐off > 9).

### 2.4. Speech Analysis

**Figure 1:**
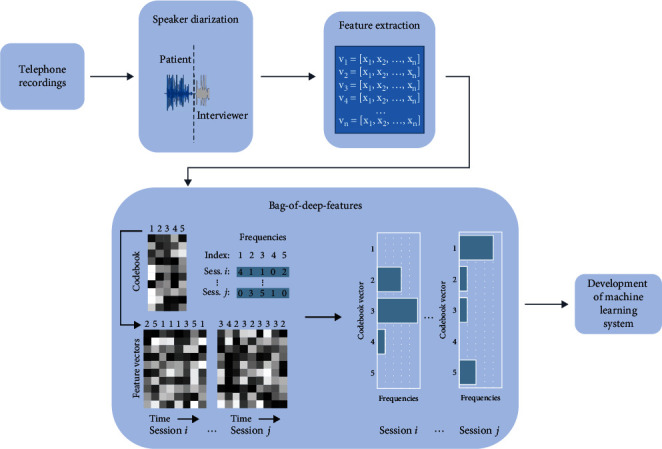
Steps of preparing and extracting speech features.

Audio recordings of telephone-based HRSD interviews were used to train the machine learning-based PSP system. To record and analyze the speech recordings, several processing steps were performed. An overview is depicted in Figure 1.

#### 2.4.1. Telephone Recordings

The telephone-based HRSD interviews were recorded via a Voice over Internet Protocol system developed by Sipgate GmbH. The interview was recorded as a single channel 32-bit.mp3 file sampled at 8 kHz.

#### 2.4.2. Speaker Diarization

As the raw interview material contained recordings of both patient and interviewer, respectively, as a first processing step, speaker diarization was performed to obtain only patient data. Due to the size of the dataset, automatic diarization was applied based on a speaker verification method presented in Dawalatabad et al. [65]. Specifically, whisperX's [66] implementation was utilized.

#### 2.4.3. Feature Extraction

In the first step, we extracted deep representations utilizing a pretrained wav2vec model, specifically a variant of wav2vec2.0 fine-tuned for emotion recognition from speech [67]. Consequently, multidimensional feature vectors containing extracted speech parameters represent the speech signals.

#### 2.4.4. Bag-of-Deep-Features

The next processing steps followed a Bag-of-Deep-Features approach, which reduces data to facilitate processing in machine learning and minimize noise [68]. The feature vectors were aggregated based on their distance in multidimensional space. Then, a representative set of feature vectors (so-called codebook vectors) were randomly sampled and saved to a codebook. In a process called quantization, feature vectors from the segments of each interview were then assigned to their nearest representation in the codebook. The number of assignments to each codebook vector was recorded in a sparse histogram representation of the interview. As the recordings vary in duration, the histograms were normalized based on their number of speech segments. The Bag-of-Deep-Features were generated on a per-fold basis (i.e., for each of the 10 folds in the validation setup). The codebooks were sampled only from vectors in the respective training partitions, ensuring zero information leakage from each fold's test partition during model training.

### 2.5. Development of the Machine Learning System

A support vector machine (SVM) with linear kernel was applied to classify the diagnostic status of MDD from the features extracted in previous steps. This model uses hyperparameters that allow the adaptation of an algorithm in the learning process of machine learning to generate an optimal model. It generates a model aimed at separating feature vectors of different classes by a maximum margin hyperplane. The adjustment of the hyperparameters affects the threshold for the classification decision. In the initial training phase, machine learning received input from both labels (diagnostic status) and features (speech parameters extracted from spectrograms) and generated a model with hyperparameters optimized for the dataset. Specifically, the SVM's complexity parameter was tuned on a logarithmic scale from 1 to 10^−6^. Furthermore, we optimized the weight assigned to depressive samples, either by inverse scaling according to class frequencies in the training data (balancing the influence of depressive and nondepressive samples) or by choosing a fixed integer from {2, 3, 4, 5}. In the evaluation phase, the model was evaluated on a dataset with only features as input. Our machine learning approach was evaluated with speaker independent 10-fold cross-validation. To evaluate our system, we report the mean and standard deviation of the balanced accuracy scores achieved on each of the ten folds. Nested random 5-fold cross-validation on each fold's training partition was used to optimize model hyperparameters based on the same metric. We make use of the scikit-learn [69] implementations for the linear SVM and cross-validation procedures.

For the additional machine learning experiments using depression scales, we utilized the same classifier, inputting item scores of the PHQ-9, HRSD17, and QIDS-C, respectively. Finally, both approaches were combined in a late fusion setting by stacking the outputs of both models and using them as input to another linear SVM classifier.

### 2.6. Implementation Details

The code for the machine learning experiments conducted in this study is publicly available on GitHub (https://github.com/mauricege/MDD-from-PSC). We used Python for the implementation and built it exclusively on open-source packages, mainly scikit-learn. A full list of dependencies can be found in the repository.

### 2.7. Statistical Analysis

In order to evaluate PSP-based classifications of diagnostic status against those of state-of-the-art instruments, sensitivity (i.e., number of true positives/(number of true positives + number of false negatives)), specificity (i.e., number of true negatives/(number of true negatives + number of false positives)), and balanced accuracy (i.e., (sensitivity + specificity)/2) of machine learning-based classifications and classifications based on cut-off scores from HRSD, QIDS-C, and PHQ-9 were calculated. Throughout all analyses, the SCID served as validation criterion. Then, balanced accuracy, sensitivity, and specificity were tested against chance level using a permutation test [70]. For the permutation test, we generated 100 resamples with permuted labels. For each of the 100 resamples, we ran SVM classifications to generate a nonparametric distribution of performance metrics (i.e., balanced accuracy, sensitivity, and specificity). We tested statistical significance by determining if the balanced accuracy of the PSP system exceeded the null distribution at a significance level of *α* = 0.05.

## 3. Results

Sociodemographic characteristics at baseline are shown in Table 1. Across all time points and conditions, 28.0% of the datasets came from patients who were diagnosed with MDD at the time of the interview according to the respective SCID.

**Figure 2:**
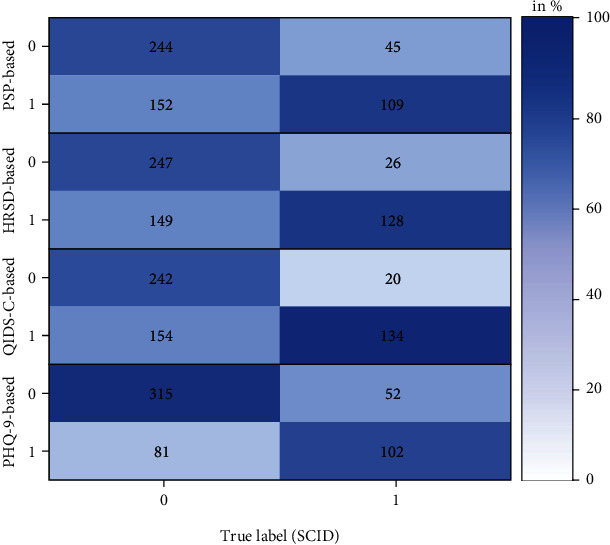
Confusion matrix of diagnostic status classification with SCID as criterion with the number of individuals classified into the respective category. SCID = Structured Clinical Interview for DSM-IV; PSP = paralinguistic speech processing; 0 = currently in remission; 1 = currently depressed. Percentages in the legend refer to the ratio of correct and incorrect predictions within the true label.

**Figure 3:**
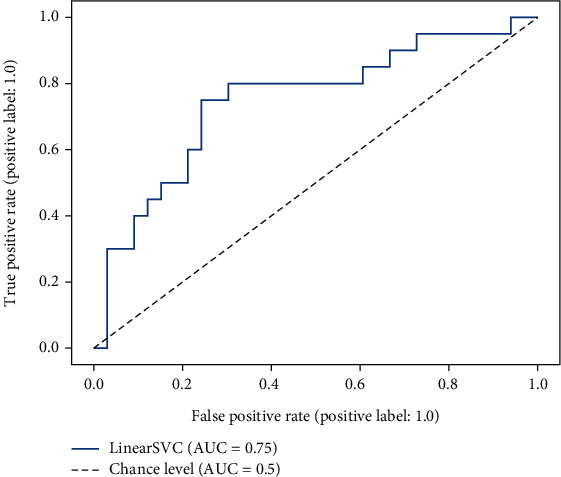
Receiver operating characteristic curve from a representative fold (#7) of the PSP system's cross-validation. SVC = support vector classification; AUC = area under the curve.

Across folds, the mean balanced accuracy of the PSP-based classifications was 65.91% (SD = 0.05), with a mean sensitivity of 69.72% (SD = 0.09) and a mean specificity of 62.11% (SD = 0.07). Performances achieved in the individual folds are shown in Table 2. A confusion matrix of PSP-based classifications and classifications based on cut-off scores in the HRSD, the QIDS-C, and the PHQ-9, respectively, is depicted in Figure 2. Performances of the PSP system for women, men, and different age groups, respectively, are shown in Table 3. Further, a receiver operating characteristic curve for one exemplary fold is shown in Figure 3.

**Figure 4:**
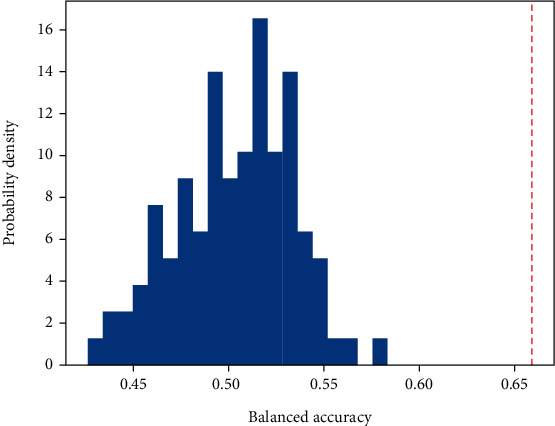
Nonparametric distribution of balanced accuracy from permuted resamples. The dotted red line refers to the mean balanced accuracy of our PSP system.

Table 4 shows the balanced accuracy, the sensitivity, and the specificity of diagnostic status classification based on the cut-off scores of HRSD, QIDS-C, and PHQ-9 with the validation criterion being diagnostic status according to SCID. The nonparametric distribution of balanced accuracy obtained by classifying permuted resamples is shown in Figure 4. The permutation test indicates that the balanced accuracy of the PSP system is significantly better than chance (*p* < 0.001). The same applies to sensitivity (*p* < 0.001) and specificity (*p* < 0.001). Performance metrics of SVM classifications based on depression scale items only and their fusion with speech analysis are shown in Table 5.

## 4. Discussion

The primary goal of our study was developing and evaluating a PSP system to classify the diagnostic status of MDD from 550 naturalistic speech samples from 267 patients in routine clinical care. The PSP system developed in our study could classify diagnostic status as assessed with the SCID with a balanced accuracy of 66%, a sensitivity of 70%, and a specificity of 62%. Permutation tests indicated that the PSP-based classifications are significantly better than chance level, showing that the PSP system is able to detect patterns meaningful for MDD. Our PSP system thus constitutes a significant proof-of-concept. Furthermore, performance metrics suggested higher sensitivity/lower specificity for the youngest (aged 18–29; sensitivity: 92%, specificity: 48%) and the oldest (aged 60+; sensitivity: 88%, specificity: 50%) patients compared to the age groups in-between (sensitivity: 65–70%, specificity: 62–65%). We further found a gender bias with higher sensitivity for women (73%) compared to men (67%) and lower specificity for women (57%) compared to men (68%). Notably, the performance of the PSP system does not reach the accuracy of common depression scales. In addition, fusing depression scales with speech analysis did not improve classification performance compared to classifying MDD with depression scales alone. This shows that speech analysis offers no advantage in the classification of MDD over traditional classification based on cut-off scores and automated classification based on depression scales.

It is of note that the accuracy of the PSP system developed in this study is notably lower than the accuracy of some PSP systems reported in the literature (primarily with a maximum balanced accuracy of 91% in a sample from the general population [36] and with a maximum balanced accuracy of 77% in a clinical sample [44]). However, it needs to be acknowledged that the current study aims to overcome some problems associated with previous research that likely lead to higher accuracy but at the same time to lower ecological validity if used for clinical purposes. First, several studies developing PSP systems to detect depression exclusively used nonclinical samples (e.g., [36, 51]). Therefore, it is unclear to what extent the findings of these studies can be generalized to clinical populations. To overcome this limitation, we included patients in routine clinical care, which is a highly relevant target group for monitoring [10]. Second, other studies trained and evaluated their PSP systems by having them distinguish between a healthy and a clinical sample (e.g., [40, 47, 49]). However, this sample selection excludes the recruitment of participants with elevated or residual depressive symptoms, who do not meet the full criteria for a diagnosis of MDD. Therefore, such PSP systems may not be generalizable to populations in clinical care that typically include a significant number of patients with subclinical depressive symptoms, for whom monitoring is particularly relevant [71]. To amend this limitation, we used a naturalistic sample of patients in routine clinical care. Therefore, our dataset includes patients meeting criteria for MDD and patients no longer meeting criteria for MDD with and without residual depressive symptoms, increasing generalizability of the PSP system in a clinical population. Third, various studies exclusively used the cut-off scores of self-report instruments that are associated with problems such as memory and reporting biases [72, 73] as indicators of the presence/absence of a diagnosis of MDD (e.g., [36, 44, 52]). Thus, in the present study, we used the SCID as the gold standard for diagnosing MDD [60] and complemented it with well-established interviews and a self-report measure. Fourth, many studies used comparatively small sample sizes that are unlikely to produce reliable results [42–44, 74]. To overcome this limitation, we used a considerably larger sample. Fifth, several previous studies trained their PSP systems with speech recorded under laboratory conditions (e.g., [42, 47, 56]). Thus, it is unclear whether findings from these studies can be generalized to real-world recording conditions. Evidence for limited generalizability across recording conditions comes from a study showing that the same machine learning approach reached a balanced accuracy of 91% when using laboratory-recorded speech compared to 72% when using speech recorded in the field [36]. To overcome this limitation, we used naturalistic speech to increase the PSP system's applicability to real-world monitoring settings. Arguably, speech recorded from telephone interviews is a relevant data source for monitoring, since telecommunication between patients and clinical care providers has grown rapidly in recent years [75]. Sixth, many studies generated speech samples by having participants complete phonetic tasks or respond to open questions (e.g., [44, 45]). This procedure is associated with additional effort on the participant's side, which may lead to low adherence rates to a monitoring application [11]. Thus, we analyzed telephone interviews that are used in various settings for clinical purposes. Utilizing these interviews to extract additional information on diagnostic status with the help of speech analysis does not require additional effort by patients and clinicians. Finally, most previous studies applied a variety of machine learning methods (with substantial variability in included features) on the same datasets when developing PSP systems. Whereas this improves the likelihood of developing one or more systems that achieve good accuracy, it likely overestimates the accuracy, since methods often do not work equally well across datasets (e.g., in [36], using the same method achieved a balanced accuracy of 91% in one dataset and only one of 65% in another dataset). Since our aim was to evaluate the applicability of a PSP system in clinical practice, we decided on using only one deep learning approach as a state-of-the-art machine learning method with high generalizability. In summary, the present study contributes significantly to the literature, as it provides a proof-of-concept that the PSP-based assessment of MDD can be applied to naturalistic speech samples from individuals who had all been previously treated for MDD and when the gold standard of diagnosing MDD is used.

From a practical perspective, the findings from the present study must be evaluated more critically. Pettersson et al. [76] proposed a sensitivity of 80% and a specificity of 70% to be acceptable for case identification. Both, a lack of treatment for false-negative cases and unnecessary follow-up treatments for false-positive cases, may lead to negative outcomes for those affected. Accordingly, the risk of false negatives cannot be considered acceptable in our PSP system with a sensitivity of 70%. The same applies to the risk of false positives, which is unsatisfactory with a specificity of 62% in our PSP system. These metrics mean that in a group of 100 formerly depressed patients with a recurrence rate of 30% [77], our PSP system would falsely classify nine currently depressed patients as nondepressed. Further, from the 42 patients it would classify as currently depressed, 21 patients would actually be nondepressed. In comparison, the PHQ-9 would result in 10 patients being falsely classified as nondepressed and 14 patients being falsely classified as currently depressed. Findings suggest that our PSP system would not outperform one of the shortest screening tools for depression (conducting and interpreting the PHQ-9 takes about five minutes). While the advantages of a time-efficient and cost-effective measure apply to a similar extent to PSP systems and the PHQ-9, the higher accuracy of the latter means that PSP systems do not yet pose a real alternative for clinical practice. However, further appeal of PSP systems, namely, the nonintrusive, automated, and objective assessment of MDD, nevertheless urges future research to improve the performance and generalizability of speech analysis methods by training models with naturalistic samples and reliable clinical data. Another finding relevant for clinical practice is the apparent age and gender bias in classifications. Our results suggest that the PSP system shows higher sensitivity and lower specificity in younger (aged 18–29) and older (aged 60+) patients, leading to a potential overdetection of MDD in these age groups. Further, we found higher sensitivity and lower specificity in women compared to men, potentially leading to an overdetection of MDD in women and/or an underdetection in men. Such bias in clinical practice may lead to significant individual and public health consequences [78, 79]. There are a number of potential causes that may have been relevant for these biases, such as variations of emotion expression in gender [80] and age groups [81], different symptom presentation in gender [82] and age groups [83], differences in validity of depression assessments for gender [84] and age groups [85], and different vocal characteristics for gender [86] and age groups [87]. Importantly, such factors potentially contribute to biased classifications in both common depression scales and PSP systems. In addition to the above-mentioned general causes for gender and age differences in (voice-based) depression assessments, bias may have resulted from factors specific to this study: a greater proportion of current MDD diagnoses within the measurement time points of female participants (31% compared to 27% in male participants) may have led to a model bias that increased the probability of classifying women as currently depressed. Further, there were fewer participants in the youngest and oldest age groups than in other age groups, which may have led to less reliable results. For machine learning approaches, methods have been developed that have the potential to diminish bias (e.g., the application of gender- [46, 47] and age-dependent [88] models or the development of separate models for demographic groups [48]). However, it has not yet been possible to completely eliminate bias through age- and gender-dependent models. In studies applying such methods, performance differences between age groups [88] and gender [46, 47] were not substantially lower than in the present study. Thus, future studies should identify specific causes of bias and further work on improving methods that increase the fairness of PSP systems. Future studies that apply methods that effectively eliminate bias may be able to develop PSP systems that exceed the objectivity and validity of the current approach as well as present depression assessment methods.

Next to the aspect of potential age and gender biases, the following limitations have to be considered: First, the generalizability of our PSP system to other recording conditions and other populations is unclear. Thus, future studies should test our PSP system under different recording conditions and in other populations (e.g., nonclinical samples) to test its generalizability. Second, we used audio recordings of the HRSD for the development of the PSP system. Due to the nature of this interview, currently nondepressed patients are likely to negate more answers than currently depressed patients, which may have had an influence on speech analysis. However, the HRSD includes open-ended responses, and in each interview from our sample, at least one question was negated. Thus, we do not expect an effect of the responses' content on the classifications of the PSP system. However, future research should verify this assumption by comparing PSP systems trained on various audio recordings, including but not limited to clinical interviews. Third, for this study, we only predicted diagnostic status and not depressive symptom severity (as a continuous variable), although the latter is particularly relevant in the context of individual symptom monitoring. The decision to focus on this particular outcome was based on the fact that the categorical information on the presence or absence of a diagnosis is critical for clinical decision-making in routine healthcare (for example, with regard to whether or not the healthcare system pays for treatment [15]). Nevertheless, future studies should also develop and evaluate machine learning approaches assessing depressive symptom severity as a continuous variable. Ideally, these studies should also work to clarify whether symptoms of MDD differ with respect to the extent to which they can be assessed with PSP systems. Finally, our PSP system does not provide explanations about the causal mechanisms between MDD and speech. Thus, future studies should work to clarify how speech affects other symptoms of depression (e.g., with the help of network analyses or experimental studies).

## 5. Conclusions

Overall, our study has made important contributions in researching applications of speech analysis with machine learning in the context of routine clinical care. It is the first study to evaluate the validity of automated monitoring of diagnostic status of MDD with external validation criteria in a naturalistic speech sample. In addition, our study aimed for generalizability with its large clinical sample, the naturalistic setting to record speech, and the deep learning approach. Results suggest that our PSP system is not sufficiently accurate for an exclusive use in clinical practice. By applying state-of-the-art methods for developing the PSP system, we can show that the current state of automated depression detection with speech analysis is not yet ready for practical application in clinical practice. We recommend that future research on speech analysis prioritizes integrating the practicalities and requirements of clinical practice. There is a need to develop machine learning methods that enable accurate classifications based on naturalistic samples and reliable clinical data and that have been externally validated with state-of-the-art depression assessment methods.

## Figures and Tables

**Table 1:** Sample characteristics.

Variable	All participants (*N* = 267)
Age, *M* (SD)	45.6 (10.5)
Sex, female (%)	59.2
School education (%)	
<9 years	0.8
9 years	10.0
10 years	27.3
>10 years	61.9
Nationality (%)	
German	98.5
Other	1.5
Family status (%)	
Single	20.1
Married/living in a partnership	58.3
Separated/divorced	19.3
Widowed	2.3
Current occupation, yes (%)	78.8
HRSD	
Baseline, *M* (SD)	9.58 (5.94)
Overall, *M* (SD)	8.89 (6.91)
QIDS-C	
Baseline, *M* (SD)	7.09 (4.24)
Overall, *M* (SD)	6.80 (5.12)
PHQ-9	
Baseline, *M* (SD)	8.43 (4.42)
Overall, *M* (SD)	7.77 (5.12)

HRSD = Hamilton Rating Scale for Depression; QIDS-C = Quick Inventory of Depressive Symptomatology–Clinician-rated; PHQ-9 = depression module of the Patient Health Questionnaire.

**Table 2:** Accuracy, sensitivity, and specificity for individual folds.

Fold #	Balanced accuracy	Sensitivity	Specificity
1	0.74	0.81	0.67
2	0.65	0.65	0.65
3	0.63	0.67	0.60
4	0.70	0.77	0.63
5	0.59	0.70	0.48
6	0.63	0.75	0.50
7	0.65	0.62	0.69
8	0.75	0.80	0.70
9	0.66	0.71	0.60
10	0.60	0.50	0.70

**Table 3:** Accuracy, sensitivity, and specificity of the PSP system for women, men, and different age groups.

Variable	*N*	Balanced accuracy	Sensitivity	Specificity
Gender				
Women	323	0.65	0.73	0.57
Men	227	0.67	0.67	0.68
Age group				
18–29	45	0.70	0.92	0.48
30–39	72	0.63	0.65	0.62
40–49	205	0.67	0.70	0.65
50–59	190	0.65	0.67	0.64
60+	38	0.69	0.88	0.50

**Table 4:** Accuracy, sensitivity, and specificity of classifications based on the PSP system and based on cut-off scores from depression scales.

Measure	Balanced accuracy	Sensitivity	Specificity
PSP-based	0.66	0.70	0.62
HRSD score	0.73	0.83	0.62
QIDS-C score	0.74	0.87	0.61
PHQ-9 score	0.73	0.66	0.80

Interview and questionnaire data across time points. PSP = paralinguistic speech processing; HRSD = Hamilton Rating Scale for Depression; QIDS-C = Quick Inventory of Depressive Symptomatology–Clinician-rated; PHQ-9 = depression module of the Patient Health Questionnaire.

**Table 5:** Machine learning classifications based on depression scale items.

	Balanced accuracy mean (SD)	
	Exclusive model	Fused model
HRSD-based	0.72 (0.07)	0.73 (0.07)
QIDS-C-based	0.75 (0.07)	0.76 (0.07)
PHQ-9-based	0.73 (0.07)	0.73 (0.06)

The exclusive models only use scale items, whereas the fused models combine audio features and scale items. HRSD = Hamilton Rating Scale for Depression; QIDS-C = Quick Inventory of Depressive Symptomatology–Clinician-rated; PHQ-9 = depression module of the Patient Health Questionnaire.

## Data Availability

The programming code for this study is available at this link: https://github.com/mauricege/MDD-from-PSC.
